# The Irony of Iron: The Element with Diverse Influence on Neurodegenerative Diseases

**DOI:** 10.3390/ijms25084269

**Published:** 2024-04-12

**Authors:** Seojin Lee, Gabor G. Kovacs

**Affiliations:** 1 Tanz Centre for Research in Neurodegenerative Diseases, University of Toronto, Toronto, ON M5T 0S8, Canada; lseojin.lee@mail.utoronto.ca; 2 Department of Laboratory Medicine and Pathobiology, University of Toronto, Toronto, ON M5S 1A8, Canada; 3 Edmond J. Safra Program in Parkinson’s Disease, Rossy Program for PSP Research and the Morton and Gloria Shulman Movement Disorders Clinic, Toronto Western Hospital, Toronto, ON M5T 2S8, Canada

**Keywords:** neurodegeneration, iron, tau, alpha-synuclein, amyloid-beta

## Abstract

Iron accumulation in the brain is a common feature of many neurodegenerative diseases. Its involvement spans across the main proteinopathies involving tau, amyloid-beta, alpha-synuclein, and TDP-43. Accumulating evidence supports the contribution of iron in disease pathologies, but the delineation of its pathogenic role is yet challenged by the complex involvement of iron in multiple neurotoxicity mechanisms and evidence supporting a reciprocal influence between accumulation of iron and protein pathology. Here, we review the major proteinopathy-specific observations supporting four distinct hypotheses: (1) iron deposition is a consequence of protein pathology; (2) iron promotes protein pathology; (3) iron protects from or hinders protein pathology; and (4) deposition of iron and protein pathology contribute parallelly to pathogenesis. Iron is an essential element for physiological brain function, requiring a fine balance of its levels. Understanding of disease-related iron accumulation at a more intricate and systemic level is critical for advancements in iron chelation therapies.

## 1. Introduction and Objectives of This Review

Brain iron disbalance is associated with a wide spectrum of neurological disorders, including stroke [1,2], mental disorders [3], and cancer [4], reflecting its comprehensive involvement in human brain physiology. Additionally, post-mortem examinations and patient MRI observations have revealed abnormal accumulation of brain iron in a group of neurodegenerative diseases, including, but not limited to, Alzheimer’s disease (AD), Parkinson’s disease (PD), multiple system atrophy (MSA), and progressive supranuclear palsy (PSP) [5]. These diseases are also discussed as neurodegenerative proteinopathies, referring to a group of diseases associated with the deposition of misfolded proteins in the brain. The involvement of iron in disease pathogenesis varies at large by the associated anatomical regions and the severity of its deposition. Advancements in iron-sensitive magnetic resonance imaging (MRI) now allow the pathologic iron burden in patients to aid disease diagnosis and prognosis. However, the comprehensive involvement of iron in multiple physiological and pathological brain functions challenges our understanding of the functional relevance of iron accumulation in disease pathogenesis.

**Figure 1:**
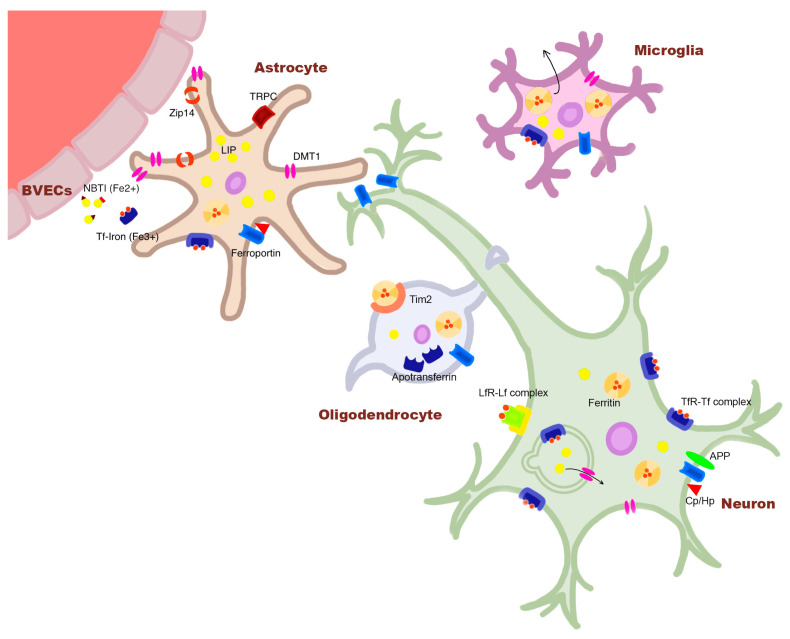
Distinct iron homeostasis in cell types of the central nervous system (CNS). Once iron enters the brain interstitial fluid (ISF) through the blood–brain barrier consisting of brain vascular endothelial cells (BVECs), iron exists in two forms: ferric iron (Fe^3+^) bound to Tf (holo-Tf) and ferrous iron (Fe^2+^) bound to ATP or citrate (non-transferrin-bound iron; NTBI). Iron is taken up at the astrocytic end-feet predominantly via DMT1s but also by metal transporter ZIP14 and transient receptor potential canonical channels. Inside the cytosol, iron is either stored bound to the major cellular iron-storage protein ferritin in the ferric form, or it exists as a pool of ferrous iron referred to as labile iron pool (LIP). In neurons, iron enters mostly through the Tf cycle in which, upon binding of holo-Tf to Tf receptor (TfR) on the membrane, the TfR–Tf complex is internalized via formation of clathrin-coated pit. Iron is released from Tf by endosomal proton efflux and exits into the cytoplasm through DMT1s. Iron can also enter neurons through DMT1s, to a lesser degree, and lactoferrin receptors (LfR), which ferric-iron-bound lactoferrin (Lf) binds for internalization. In oligodendrocytes, ferritin can be taken up through the membranous protein, T-cell immunoglobin and mucin-domain-containing protein-2 (Tim2), and it is responsible for the synthesis and release of Tf proteins into the ISF. In microglia, iron enters the cell through DMT1s and TfRs, and it releases ferritin into the CNS. Universally across the cell types, iron is exported out into the ISF by a membranous protein, ferroportin. In neurons, tau-mediated APP transport to the membrane stabilizes ferroportin onto the membrane and aids iron export. Modified from Lee et al. [9].

Brain iron is an essential element for human brain physiology, existing in two forms: ferric (Fe^3+^) and ferrous (Fe^2+^) iron. Ferric iron is found in the extracellular space bound to transferrin (Tf) and in cell bodies bound to the iron-storage protein, ferritin. Ferrous iron, on the other hand, is found mostly free both inside and outside of cells (non-transferrin-bound iron; NTBI), constituting the cellular labile iron pool [6]. The latter species, upon accumulation, contributes to the production of toxic reactive oxygen species (ROS) via participation in Fenton reaction [7,8]. Brain iron homeostasis is independent from the systemic concentrations of the circulatory system due to the blood–brain barrier [6]. Its levels are tightly controlled, as a slight increase may lead to detrimental effects on neuronal health. Importantly, such regulation is a dynamic system, which comprises distinct homeostatic pathways in different cell populations, involving unique regulatory proteins favoring different iron species (Figure 1). Therefore, for a comprehensive delineation of pathogenic iron functions in disease pathogenesis, iron dysregulation needs to be studied at the cellular and cell-type-specific levels.

In this review, we carefully examine the pathogenic role of abnormally accumulating iron in the pathogenesis of AD (combined amyloid-β, Aβ, and tau proteinopathy), PSP (tauopathy), and furthermore, PD and MSA (both α-synucleinopathies). Although AD and PSP, as well as PD and MSA, show overlapping involvement of the same misfolded protein pathology, they present with distinct cellular and anatomical vulnerability patterns. In a disease-specific manner, we summarize recent literature evaluating the regional and cellular pathological dysregulation of iron and address the popular question in the literature: Is iron a driver or consequence of protein pathology? We review existing evidence supporting the following distinct hypotheses: (1) iron accumulation is a consequence of pathological alterations in protein; (2) iron accumulation promotes protein pathology; (3) iron accumulation protects from or hinders the accumulation of misfolded protein pathology; and (4) iron dyshomeostasis and protein accumulation are parallel and converging pathways.

## 2. Alzheimer’s Disease

AD presents with sporadic and familial forms, the latter most commonly associated with mutations in the amyloid protein precursor (*APP*), presenilin-1 (*PSEN1*), and presenilin-2 (*PSEN2*) genes [10]. AD is characterized clinically by progressive memory loss and cognitive decline and neuropathologically by the extracellular deposition of Aβ plaques accompanied by intraneuronal accumulation of tau pathology (neuropil threads, neurofibrillary tangles and pretangles) [11]. Different brain regions show vulnerability to these protein pathologies. Aβ is first observed to accumulate in the isocortices and tau to initially appear in the transentorhinal cortex. Aβ pathology progressively affects the hippocampus, entorhinal cortex, basal ganglia and diencephalon, brainstem, and the cerebellum, whereas tau pathology involves the entorhinal cortex, inferior and middle temporal gyri, and the occipital cortex (stage V, VI) [12,13,14]. The exact spatiotemporal association between the two protein pathologies in disease progression is not fully understood. Interestingly, there are early observations of brainstem and subcortical tau pathology, which are thought to precede cortical (including entorhinal) involvement described originally as stages by Braak and Braak [15,16,17]. Regardless, the two protein pathologies are seen to accumulate years before the clinical onset, generally with earlier deposition of Aβ, and co-propagate along disease progression.

### 2.1. Iron Dysregulation in AD

**Figure 2:**
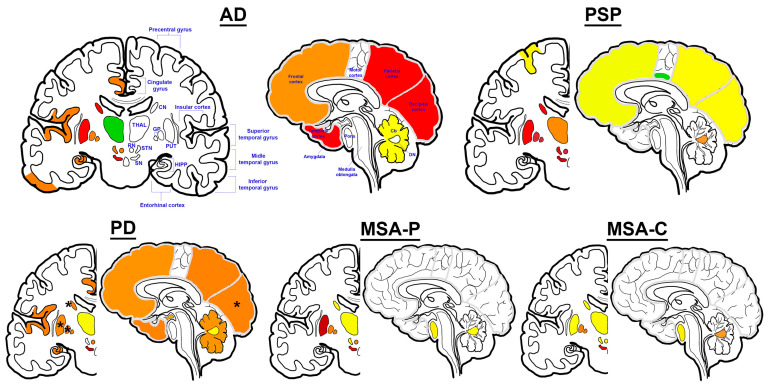
Iron deposition patterns in neurodegenerative proteinopathies. Among the examined regions (colored), the color scheme is as follows: decreased levels as reported [23,27] (green); examined, yet no difference reported (yellow); mix of increased and unchanged levels or scarce reports of increased levels (orange); frequently reported/supported increased levels (red); evidence of greatest increase in levels (dark red) compared to non-diseased aged controls (for details see text). Asterix represents regions with mixed reports of increased and decreased levels of iron compared to controls. Representations of iron deposition in MSA-P—and more especially, MSA-C—are mostly based on MRI examinations to focus on subtype-specific observations. In PSP, iron deposition has been reported in cerebral peduncles and oculomotor nerve, but it is not graphically represented in this figure. Abbreviations: Alzheimer’s disease (AD), progressive supranuclear palsy (PSP), Parkinson’s disease (PD), multiple system atrophy—Parkinsonian (MSA-P), multiple system atrophy—cerebellar (MSA-C), caudate nucleus (CN), thalamus (THAL), globus pallidus (GP), putamen (PUT), red nucleus (RN), subthalamic nucleus (STN), substantia nigra (SN), hippocampus (HIPP), cerebellum (Cb), dentate nucleus (DN).

Iron imbalance in AD-affected brains is thought to be a widespread pathological phenomenon affecting several brain areas. Increased levels of iron are found in the whole cortex, including frontal, temporal, parietal, occipital, insular, and cingulate cortices [18,19,20,21], where temporal and occipital lobes are often found with a prominent burden [20,22] (Figure 2). Subcortical regions, most consistently in the caudate nucleus and the putamen, are observed with higher iron burden, which are reported to be more closely associated with disease progression defined by advanced cognitive impairment and duration of disease [18,20,23,24]. This may explain the inconsistency in subcortical iron examinations in AD [25].

An extensive number of recent research works demonstrate a strong association between pathological iron and cognitive decline [25]. Evaluating Aβ-CSF-positive cognitively unimpaired versus impaired AD patients, quantitative susceptibility mapping (QSM) values of the cortical and inferior temporal gyrus (ITG), as well as their tau-SUVR uptake levels were found to be increased, with a negative correlation between the QSM signal in the ITG and global cognition, as quantified by the mini-mental state examination (MMSE) [19]. In longitudinal, post-mortem, and observational studies, MMSE was associated with or predicted with increased iron levels in the ITG, cingulate cortex, insular cortex, right angular gyrus, supramarginal gyrus, lateral occipital cortex, parietal operculum cortex, left frontal operculum cortex, and the inferior frontal gyrus [18,19,21]. Associations with the Montreal cognitive assessment (MoCA) scores were also observed in the right angular gyrus, supramarginal gyrus, lateral occipital cortex, superior temporal gyrus, and frontal cortices [18]. Cogswell et al. [23], on the other hand, also demonstrate the association of subcortical iron levels, rather than of cortical regions, with lower Short Test of Mental Status (STMS) cognition scores. Although further validation is required, CSF iron content is hypothesized to reflect global brain iron levels. A longitudinal study by Ayton et al. [26] evaluating CSF ferritin levels in cognitively normal, mild cognitive impairment (MCI), and AD subjects also demonstrated a correlation of baseline CSF ferritin levels with poor cognitive performance over 7 years, as measured by the Alzheimer’s disease assessment scale (ADAS-Cog13). Interestingly, baseline CSF ferritin levels were demonstrated to be significantly elevated in APOE-ε4-positive individuals and predicted MCI conversion to AD, proposing brain iron elevation as an etiological vulnerability factor for AD [26].

Post-mortem and MRI studies evaluating both iron and protein pathology loads reveal different associations of iron burden with Aβ and tau pathology. The correlation between pathological iron and tau burden is strongly supported. Positive correlations have been reported in cortices, including the temporal lobe, ITG, lingual cortex, and the amygdala, [19,20,28,29], but to a greater extent in subcortical regions, including pallidum, putamen, and the caudate nucleus [23,29]. Such correlation is also often associated with cognitive impairment and volume loss [19,23,28], supporting a strong relationship among iron accumulation, tau pathology, cognitive dysfunction, and brain atrophy in disease pathogenesis. Recent clinical trials with anti-Aβ antibodies suggest that reducing Aβ may have an impact on cognitive decline in AD patients [30]. Given the discussed association between iron, tau deposition, and cognitive dysfunction, the association of iron deposition with that of Aβ pathology is of interest. However, the correlation between iron and Aβ pathology load is rather inconsistent in the literature, showing positive [23,31,32], no [28,29], or even negative correlations [23]. Positive correlations are more frequently associated with later affected basal ganglia regions, whereas negative or absence of correlations are more frequently associated with cortical regions. In the context of protein pathology accumulation and propagation along AD progression, such evidence suggests that iron accumulation may be more closely associated with tau deposition and brain structural changes rather than with the trajectory of Aβ deposition, both temporally and in magnitude. Indeed, several post-mortem studies emphasized that tau pathology correlates more with cognitive decline in AD (for a review, see Nelson P et al. [33]).

Although post-mortem and in vivo MRI examinations provide valuable insight into the anatomical and clinical relationship between iron and the protein pathologies, it is important to note that there are limitations to these examinations, such as the use of samples from varying stages of disease (post-mortem studies are mostly limited to a more developed or end-stage disease), limited resolution threshold, possible off-target binding, and imaging or analysis parameter variance. Cellular and molecular level observations are critical for a comprehensive understanding of iron’s relationship with protein pathologies, and they are discussed in the following sections.

### 2.2. Cellular Iron Dysregulation in AD

Deconstructing the regional deposition of AD iron at the cellular level, existing evidence points to the important role of glia in handling excess iron. In the middle frontal gyrus of human post-mortem AD brains, neurons and endothelial cells (EC) showed little to no iron, similar to age-matched control brains [34]. The expression of TfR—a neuronal-predominant iron uptake protein—was seen to be generally decreased in AD-affected cortices, with a significant decline in the cerebral cortex, hippocampus, temporal, and occipital cortex [35]. On the other hand, deposition of ferritin and hemosiderin was observed in glial cells—particularly in activated microglia on the periphery of or infiltrating senile plaques—in post-mortem AD brains [36,37,38]. Importantly, such microglial population was found solely in high-pathology load subjects with Thal phase V and Braak stage V/VI, suggesting that microglial involvement of iron accumulation may occur in later stages [37]. Parallelly, exposure to Aβ was shown to elicit iron homeostatic changes in astrocytes [39], and single-cell RNA sequencing revealed ferroptosis genes to be significantly dysregulated in AD entorhinal cortex astrocytes [40], suggesting that AD-related iron dysregulation may be a dynamic process involving multiple cell types throughout disease progression. This could be a possible explanation for the lack of MRI iron level differences observed in MCI versus AD patients [21,31], as well as the persistence of iron cognition correlations without bulk changes in regional iron levels in AD patients, as will be discussed in more depth in the following sections [18,22].

In the following sections, we summarize literature data supporting the different hypotheses mentioned previously for both Aβ and tau proteins. The two protein pathologies are discussed separately for Hypotheses 1 and 2 for a comprehensive delineation of iron functions in the entire spectrum of AD pathogenesis or progression.

### 2.3. Hypothesis 1

#### 2.3.1. Iron Accumulation Is a Consequence of Pathological Alterations Related to Aβ

Physiologically, the amyloid precursor protein (APP) is involved in cellular iron homeostasis by stabilizing the iron export protein, ferroportin (FPN), on the cell membrane to facilitate iron efflux, although its role as a ferroxidase is debated by conflicting literature [41,42,43,44]. Moreover, APP inhibits heme oxygenase, which liberates iron from heme [45]. Therefore, disruptions in APP can induce alterations in cellular iron. Accordingly, inducing the amyloidogenic pathway of APP in primary neuronal culture led to greater cellular iron accumulation by reducing APP-mediated stabilization of FPN [46]. Such APP-FPN stabilization is demonstrated to be mediated by tau transport of APP to the cell membrane [42]. As tau expression is significantly higher in neurons [47], we may speculate that glia may have other mechanisms for maintaining iron homeostasis, which do not involve APP, making them relatively less vulnerable to Aβ-pathology-induced iron dysregulation. On the other hand, evidence suggests that astrocytes respond to extracellular Aβ pathology. Primary astrocytic culture from mice demonstrated increased expression of ferritin upon Aβ 1-42 exposure [48]. Moreover, Aβ exposure to iPSc-derived astrocytic culture resulted in increased iron uptake by astrocytes, as well as an increase in apo-Tf secretion, which led to a three-fold increase in iron transport across the blood–brain barrier (BBB) model of EC cells [39]. In this study, the authors also demonstrated that exposure of EC to media containing classic A cytokines did not elicit changes in iron transport, suggesting that increased iron transport was not due to reactive astrocytes but due to Aβ-induced astrocytic alterations in iron regulation. In AD-affected brains, such mechanism may be exaggerated due to the sequestration of extracellular iron by Aβ plaques [49], where a lack of available iron may signal astrocytic iron uptake at the BBB, increasing the local iron burden. Furthermore, Aβ induces changes in microglia as well, in which exposure to interferon and Aβ leads to microglial conversion into an inflammatory phenotype, showing glycolytic and iron retentive characteristics with reduced phagocytic and chemotactic functions both in vitro and in APPswe/PS1dE9 mice [50]. In another in vivo mouse model, intrahippocampal injection of Aβ oligomers resulted in increased APP expression as well as iron accumulation in the hippocampus, further supporting the relationship between Aβ and iron deposition at the systemic level [51].

#### 2.3.2. Iron Accumulation Is a Consequence of Pathological Alterations in Tau

As discussed previously, tau mediates the iron export of neurons by mediating surface trafficking of APP for APP-FPN stabilization. Accordingly, in vitro, tau knock-out showed 73% higher iron retention and relocation of APP to the cytosol [42]. In vivo, tau knock-out mice accumulated iron in the cortex, hippocampus, and the substantia nigra (SN), causing age-dependent neurodegeneration [42]. Parallel to the previous idea, such involvement of tau in neuronal iron regulation may render neurons in tauopathy-affected brains more vulnerable to iron-induced toxicity. There is yet no direct evidence supporting tau-aggregation-induced iron dysregulation related to AD neuropathological change.

### 2.4. Hypothesis 2

#### 2.4.1. Iron Accumulation Promotes Aβ Pathology

Polymorphism and mutations in *Tf*—and more recently, in hemochromatosis (*HFE*)—are one of the early identified genetic risk factors for AD, supporting the association between iron dysregulation and the development of AD [52,53]. As APP contributes to iron regulation, iron in turn regulates APP expression via the iron response protein (IRP)–iron responsive element (IRE) domain interaction at the 5′- untranslated region (UTR) of APP transcripts. At high levels of cellular iron concentration, IRPs undergo a conformational change to lose their affinity for IRE, resulting in an active translation of the APP mRNA [54,55,56]. Iron is also shown to affect the non-amyloidogenic and amyloidogenic processing of APP by regulating secretase activities. Iron is involved in the regulation of furin, where at high levels of iron, it inhibits furin expression. Inhibition of furin results in inhibition of α-secretase activity, leading to relative increase in β-secretase activity and increased soluble peptide APPβ (sAPPβ) products [57]. Accordingly, in SHSY5Y cells, iron has been demonstrated to increase β-secretase activity in a dose-dependent manner [58]. However, the role of iron in secretase activity is debated in the literature. In the cortical neuronal culture from Sprague Dawley (SD) rat embryos, FAC treatment decreases β-secretase activity and the following sAPPβ and Aβ secretion into culture media. Rather, FAC treatment leads to an increase in abnormal non-amyloidogenic processing of APP, in which α-secretase-processed sAPPα is localized into the cells rather than secreted, which may have cytotoxic effects [59]. Further complicating iron’s involvement in APP processing, such effect may have cell-type-specific nature, possibly due to different processing of APP across cell types. A study by Becerill-Ortega et al. [60] demonstrates that exposure of FAC to neurons from mouse cerebral cortex results in increased KPI-APP (pro-amyloidogenic form) expression as well as secretion of Aβ 42 into the medium, possibly by inducement of imbalance in synaptic NMDARs. Such effect was recapitulated in in vivo cortices and hippocampus in which iron-injected or -fed mice showed increased levels of KPI-APP mRNA, protein, and Aβ plaques [60,61]. On the other hand, FAC treatment on astrocytes did not show any changes in the transcript and protein expression of KPI-APP or APP695 at any concentrations [60].

The involvement of iron in the oligomerization and fibrillization of Aβ also requires further examination. Although some studies demonstrate that iron promotes Aβ aggregation [58,62], other studies show that iron decreases the formation of β-sheet fibrillar structures and produces a different “strain” of Aβ aggregates, which are more toxic [63,64]. Whether this species is relevant in human AD is yet to be evaluated. In addition, although the association of iron homeostatic gene mutations with AD shows that iron dysregulation can lead to AD-related pathological changes, whether the same holds true for the development of sporadic AD is still to be questioned. In particular, as discussed above, the literature is conflicting, but there is also a lack of significant elevations in iron in the brain regions affected early in the pathological process. Furthermore, age-related iron accumulation is speculated to assume the form of glia-favoring NTBI [65], whereas in vitro experiments demonstrating the effects of iron on Aβ processes seem to be prominent in neuronal cells.

#### 2.4.2. Iron Accumulation Promotes Tau Pathology

Consistent reports of correlations between QSM measures of iron and tau pathology in AD patients strongly support the relationship between the two phenomena in disease pathology [25]. Although the degree of off-target binding of tau PET SUVR needs to be evaluated further [25], a number of in vitro and in vivo studies support the pathogenic role of iron in tau pathology. In this section, we will focus on studies which used three-repeat (3R) tau or mixed four-repeat (4R)/3R tau, and AD mouse models, for a more specific relevance to AD. First, iron is observed to promote disease-related phosphorylation of tau. The treatment of primary neuronal culture from SD rats with ferrous iron led to increased tau phosphorylation at AT8 (Ser202/Thr205), Thr181, and Ser396 sites [66]. In vivo, iron-fed C57BL/6 mice exhibited increased levels of tau phosphorylation at multiple epitopes, including Ser202/Thr205, Thr181, and Ser396, while maintaining the total tau levels [66]. Moreover, APP/PS1 mice given an iron-rich diet exhibited an increase in tau phosphorylation at Thr205, Thr 231, and Ser396 sites in the hippocampus [67]. Deferoxamine administration alone inhibited phosphorylation at Ser396, meaning that physiological iron does not function in the phosphorylation of tau at Thr sites [67].

Iron is shown to promote aggregation of tau as well; however, the distinct contribution by ferrous and ferric iron needs further clarification. Using paired helical filaments (PHF-tau) derived from human post-mortem AD tissue, ferric iron has been shown to bind to tau in a phosphorylation-dependent manner and induce irreversible aggregation of hyperphosphorylated tau in a dose-dependent manner. Ferrous iron did not bind to AD-derived tau or induced any changes in aggregation, although it was speculated to possibly mediate conformation change [68]. On the other hand, using recombinant tau (tau-410), phosphorylation of tau was shown to disrupt ferric iron binding, and ferrous iron was shown to induce significant changes in tau conformation in an exponentially dose-dependent manner. In line with the previous study, although ferric inducement of α-helix tau structure was very slow, ferric-induced changes were irreversible compared to those induced by ferrous iron [69].

Supporting these in vitro studies, in MCI and AD patients examined for QSM and tau-SUVR, it was demonstrated that in the ITG where strongest regional QSM/tau-PET correlation was found, AD patients showed high QSM and tau-PET signals, whereas MCI patients showed high QSM but lower tau-SUVR signals. This suggests that iron elevation, which is yet to be toxic, may occur prior to the tau pathology burden [19]. Moreover, studies show cortical accumulation of iron in Braak IV stage regions in MCI comparable to that observed in AD, further suggesting an earlier involvement of iron in disease pathogenesis [21].

### 2.5. Hypothesis 3: Iron Accumulation Protects from or Hinders the Accumulation of Misfolded Protein Pathology

In imaging studies focusing on aging or AD, negative correlations between Aβ pathology and iron levels are also reported [23,25,28,70]. Whether such observations represent inhibitory mechanisms between each other remains unknown, and no experimental evidence yet supports this hypothesis.

### 2.6. Hypothesis 4: Iron Dyshomeostasis and Protein Accumulation Are Parallel and Converging Pathways

Reviewing iron’s relationships with Aβ and tau pathologies separately, although functional studies have demonstrated the ability of high levels of iron to induce alterations in Aβ, inconsistent but existing reports of iron level indifferences between MCI and age-matched controls and the lack of—or negative—correlation between regional iron and Aβ levels in patient MRI observations suggest that iron deposition may not occur prior to or be in a linear relationship with amyloid deposition in AD progression. Yet, the lack of functional studies supporting Aβ-induced iron dysregulation, together with the above-mentioned negative correlations, also leave the alternative scenario that Aβ deposition is causational for iron deposition inconclusive. The stronger relationship between tau and cognitive impairment with iron accumulation in human AD brains further complements the idea that the association between Aβ and iron deposition may be relatively multifactorial. Regardless, correlations between iron and cognitive functions in different AD brain regions exist in the absence of a significant increase in the regional iron level, even at low protein pathology stages [18,22,28]. Altogether, with evidence suggesting iron-dependent or enhanced toxicity of Aβ [71], the synergistic effects of iron and Aβ on toxicity and disease progression can be considered at still subtle levels of pathological iron. Observations of co-localization between iron and Aβ plaques at the cellular level further support this idea [72].

Two comprehensive studies examining iron deposition in AD patients with varying protein pathology loads and cognitive impairment revealed that a significant increase in iron levels of the inferior and temporal cortex is only visible in high-pathology patients with dementia compared to those without dementia, as well as those with low pathology with and without dementia [22,28]. Both studies identify strong association between iron and tau levels possibly underlying their findings. A significant increase in iron is seen to be associated more with later stages of disease progression [22,24,73], perhaps—according to our discussion—either due to prolonged effects of iron and Aβ co-existence or due to the emergence of tau. Whether tau or iron accumulation occurs first in disease pathogenesis, the two disease pathologies seem to be more closely associated with one another along disease progression [22].

### 2.7. Conclusion: Iron Dysregulation in AD Pathogenesis

In vitro and animal studies support both Hypotheses 1 and 2 rather equally in terms of iron’s relationship with Aβ and more abundantly so for Hypothesis 2 than Hypothesis 1 in terms of its relationship with tau pathology (Table 1). However, correlational observations in post-mortem or living AD patients via histology or MRI further support iron’s association with tau pathology in AD disease progression rather than that of Aβ.

There is an extensive number of MRI studies investigating iron dysregulation in AD. However, the definitions of AD and the inclusion criteria differ greatly—for example, clinical diagnosis only, clinical diagnosis with CSF biomarkers, with MRI, with protein PET, and with post-mortem neuropathological diagnosis criteria—which, taking into account the involvement of mixed proteinopathy in AD brains, challenges the fine delineation of iron dysregulation in AD pathogenesis. Moreover, observations of iron-cognition correlations without a significant increase in regional iron levels shed an insight into the possibilities: 1) re-location of iron at the cellular level, 2) the synergistic effect of protein pathology and iron on toxicity, and 3) minute increases in iron levels below the imaging threshold which may have physiological implications —further complicating the delineation. For a comprehensive delineation of the role of iron imbalance in AD pathogenesis, functional studies investigating iron dynamics together with both Aβ and tau pathology are needed; these are missing in the current literature.

## 3. Progressive Supranuclear Palsy

PSP is a primary 4R tauopathy, showing neuronal, astrocytic, and oligodendroglial tau cytopathologies in affected brain regions. In particular, tufted astrocytes distinguish PSP from other tauopathies [74]. PSP is clinically heterogenous, presenting with at least seven major clinical phenotypes based on different levels of ocular motor dysfunction, postural instability, akinesia, and cognitive dysfunction [75]. These clinical subtypes are underlined neuropathologically by varying distribution and progression of cellular tau pathology [76]. Generally, however, PSP tau pathology is observed to progressively affect the basal ganglia, subthalamic nucleus, frontal cortex, cerebellum and dentate nucleus, and the occipital cortex of diseased brains [76].

### 3.1. Iron Dysregulation in PSP

Although PSP tau cytopathologies show different anatomical vulnerability patterns across clinical subtypes, importantly, the initial tau pathology is speculated to start uniformly in subcortical brain regions [76,77,78]. However, the same subcortical brain regions—including the globus pallidus (GP), SN, and the striatum—are selectively vulnerable sites for hypoxia and aging-related iron deposition, with far more pronounced levels in PSP patients [28,56,69,70,79,80,81] (Figure 2). The review by Lee et al. [82] summarizes MRI observations of iron deposition in PSP subcortical regions, altogether demonstrating the clear involvement of subcortical iron deposition in PSP pathology. Further highlighting the prominent involvement in PSP pathology, comparative studies involving a total of 53 PSP patients consistently show the greatest levels of iron deposition in these regions of PSP-affected brains compared to other forms of Parkinsonism with subcortical iron dysregulation, including MSA and PD; these are discussed in the following sections [83,84,85]. Interestingly, these comparative studies identify the red nucleus (RN) iron MRI measures for effectively distinguishing PSP patients from those with MSA and PD. To date, only very few studies have examined cortical iron dysregulation in PSP-affected brains. In six post-mortem human PSP samples, greater iron burden via increased MRI transverse relaxation times (T2, T2*) as well as ferritin expression were observed in the cerebral peduncles compared to age-matched controls [79]. However, in vivo, PSP patients were devoid of any increase in cortical iron levels observed via MRI in 12 and 40 PSP patients [27,86]. Nevertheless, Satoh et al. [87] reported positive associations between susceptibility and flortaucipir uptake in the bilateral frontal white matter and the right fusiform region in 50 PSP patients, suggesting there may be pathology-associated changes in iron homeostasis at lower concentrations in PSP cortical regions. Interestingly, iron accumulation has also been observed along the oculomotor nerve of six PSP brains, which has not been observed in aged controls [88]. A recent study by Tanaka et al. [89] demonstrates the PSP-specific involvement of tau deposition in the cranial and spinal nerves, including the oculomotor nerve, supporting a tight association between iron and tau in PSP. Accordingly, the correlations between susceptibility and flortaucipir uptake were shown to be significantly positive in the putamen, pallidum, subthalamic nucleus, RN, and the dentate nucleus of PSP patients, and it was suggested that susceptibility measures may mediate or explain the levels of flortaucipir uptake [87].

Furthermore, regional iron accumulation has been shown to correlate with atrophy as well as clinical severity in 13 and 51 PSP-affected brains, respectively [83,90,91], further supporting its involvement in disease pathology. Accordingly, iron deposition patterns have recently been compared in different PSP clinical subtypes for a possible stratification [86,87,92]. PSP—Parkinsonism (PSP-P; *n* = 9) accounted for the greatest number of regions with significantly high iron levels compared to controls (pallidum, subthalamic nucleus, SN, RN, cerebellar dentate), followed by PSP—Richardson syndrome (PSP-RS; *n* = 20) (pallidum, SN, RN, cerebellar dentate) and PSP—postural instability (PSP-PI; *n* = 4) (pallidum, SN), whereas PSP—progressive gait freezing (PSP-PGF; *n* = 7) did not exhibit any differences in all regions. Across the subtypes, significant differences were observed in the RN and the cerebellar dentate nucleus, with PSP-RS and PSP-P subtypes presenting with higher levels than other subgroups [86]. Iron dysregulation in 15 PSP-P and 14 PSP-RS patients was found to be comparable in another study as well [92]. Supporting the possible relationship of the observed subtype-specific iron differences to varying tau pathologies, 19 PSP-RS patients’ brains showed a strong correlation with iron and tau levels in the cerebellar dentate, and 10 PSP-P patients’ brains showed a strong correlation with iron and tau levels in the putamen, subthalamic nucleus, and the red nucleus [87]. Such observations suggest that cortical alterations in the iron levels may be present at the early stages of cortical variants [86].

### 3.2. Cellular Iron Dysregulation in PSP

The association between iron and PSP tau pathology is also prominent at the cellular level. In vitro, iron binds to recombinant tau proteins, and accordingly, ferritin co-localizes with tau filaments in human post-mortem PSP brains [69,93]. Such co-localization with ferritin and tau is observed mostly in glial morphology and significantly less in neurons [93]. Parallelly, the ferritin burden (%ROI) in iron-rich SN, RN, GP, and putamen of PSP brains is lower compared to controls, suggesting that the pathological form of iron occurs in non-transferrin-bound iron (NTBI) state, whose uptake is favored by glia [79]. For a further comprehensive evaluation of the PSP iron burden in different cell types, we recently mapped the subcortical iron deposition in astrocytes, oligodendrocytes, neurons, and microglia in nine post-mortem human PSP brains, and we examined their association with the cellular tau pathology. Uniformly in the examined GP, putamen, and SN, both ferrous and ferric iron were predominantly localized to astrocytes, with a strikingly high association with the cellular tau pathology. In detail, 74.5% and 65.3% of tufted astrocytes showed abnormal iron deposition in the very early affected GP and SN, respectively, and much less accumulation of iron was observed in the tau-affected oligodendrocytes and neurons [65]. Importantly, the iron burden was intensified in perivascular astrocytes, whereas aging-related microbleeds led to iron deposition predominantly in the microglia. Together with findings that astrocytic PSP tau pathology accumulates centrally around the blood vessels [94], a pathogenic relationship between iron and tau deposition seems to be heavily involved in the early pathogenesis of PSP more than any other tauopathies. Accordingly, single-nucleus RNA sequencing of the subthalamic nucleus of three PSP brains revealed increased ferroptosis signaling pathway in PSP astrocytes and oligodendrocytes [95].

Currently most studies reporting on PSP and iron are in the frame of Hypothesis 2, as detailed above.

### 3.3. Hypothesis 2: Iron Accumulation Promotes PSP-Related Tau Pathology

We highlighted above that in AD, a strong relationship between iron and tau pathology is speculated. Such relationship is even further augmented in PSP brains at the regional and cellular levels. Section 2.3.2 and Section 2.4.2 above describe in vitro and in vivo AD studies demonstrating the molecular relationship between iron and tau deposition in the context of Hypotheses 1 and 2, whose findings may be relevant to PSP as well. Specifically, in the context of PSP—a 4R tauopathy—a study using recombinant 4R2N tau revealed ferric-iron-specific inducement of tau oligomerization and fibrils in a dose-dependent manner, both with and without heparin. Moreover, the binding affinity of ferric iron was observed to be four times greater than that of ferrous iron [96].

In a retrospective study of a Parkinsonism cohort involving six PSP patients, the apparent transverse relaxation rate (R2*) and QSM measurement at an average interval of 23.6 months prior to death were correlated with post-mortem neuromelanin-positive neuron, α-synuclein, tau, and glial density (% area). Interestingly, both R2* and the QSM measurement predicted tau and glial density but not that of α-synuclein or NM+ neurons, further supporting the pathogenic relationship between iron and tau pathology in human brains [97].

### 3.4. Conclusion: Iron Dysregulation in PSP Pathogenesis

In vitro and in vivo functional studies demonstrating the possible role of iron in inducing tau pathology were discussed in detail in the previous section (Section 2.4.2), together with 4R-specific evidence above (Table 1 and Table 2). Together with human post-mortem and patient MRI observations reporting strong correlation between tau pathology and iron deposition in early affected regions—which extends to the cellular and molecular levels via the high co-localization levels of cellular iron and tau cytopathology—the current evidence points to the possible role of iron dysregulation in PSP disease etiology or early disease progression.

## 4. Parkinson’s Disease

PD is the main form of α-synucleinopathy, characterized by the accumulation of neuronal α-synuclein pathology in the form of Lewy bodies (LB) or Lewy neurites and the marked loss of dopaminergic neurons in the SN pars compacta (SNpc) [98]. Accordingly, PD is clinically distinguished by the presentation of primary motor features, including bradykinesia, rest tremor, rigidity, and loss of postural reflexes [99]. Generally, α-synuclein pathology is observed to affect the medulla oblongata, pons, mesencephalon (particularly SN), limbic areas, and the neocortical areas in a sequential manner [100], although initial vulnerability of the olfactory bulb and the enteric nervous system is discussed [101].

### 4.1. Iron Dysregulation in PD

Since the early examination of iron deposition in the SN of human post-mortem PD brains [102], subcortical iron deposition has been extensively studied in PD animal models, patients, and post-mortem human sample material as a potential disease hallmark. However, compared to the subcortical iron burden in MSA and PSP, iron accumulation in PD has been shown to be relatively less pronounced in the focused nuclei. Even in the SN where iron deposition is greatest in PD whole brains, the levels are observed to be comparably lower [85,103,104,105]. Moreover, MRI observations of iron deposition in the striatal regions of PD patients are inconsistent; they vary by reports of increased susceptibility in the putamen, GP, and the caudate nucleus [24,106,107,108,109] or reports of significant indifference [84,105,108,110,111,112,113] compared to healthy controls (Figure 2). Select studies even report significantly decreased iron levels in the putamen [114] and the caudal motor striatum [110] compared to age-matched controls. These observations suggest iron accumulation in pathology-related regions in PD may be a dynamic process throughout disease progression, particularly considering the different duration of diseases of PD patients across studies [84]. Accordingly, the evaluations of CSF iron and ferritin in PD patients report non-significant differences with those of healthy controls [115,116]. On the other hand, pathological iron accumulation in the SN was most consistently reported [24,106,107,108,109,111,113,114,117] and ignited nigral-focused subregional and temporal examinations [118,119,120,121].

Interestingly, many studies report a correlation between subcortical iron levels and the severity of motor symptoms in PD patients, demonstrated by positive correlations between the Movement Disorder Society—Unified Parkinson’s disease rating scale-III (MDS-UPDRS-III) scores and increased QSM or R2* values in the putamen [106,107], GP [107,122], and the SN [117,122,123]. Correlations between nigral iron levels and motor scores are observed even in patients in middle to later stages (Hoehn–Yahr stage > 2, or duration of disease > 5 years) [117,122,123] beyond the timepoint of dopaminergic neuronal depletion, suggesting that the iron in these regions may have additional toxicity mechanisms other than neuronal cytotoxicity. On the other hand, increased cortical iron levels have been found to correlate with cognitive impairment. In the middle to more advanced stages of PD patients, the levels of iron in the frontal, posterior parietal, insular, and temporal cortices were found to be increased compared to age-matched controls [106,124]. The bilateral increase in absolute susceptibility in the hippocampus, thalamus, prefrontal cortex, basal forebrain, and rostral caudate nucleus correlated with decreasing MoCA, as well as with dementia risk score in bilateral increases in prefrontal, frontal, cingulate, temporal, parietal, medial occipital cortex, and basal forebrain [106]. Correlations with MoCA and Open Essence are also reported with increased iron levels in the cuneus, amygdala, and fusiform gyrus [107]. Such correlation may represent iron toxicity associated with the spread of α-synuclein pathology from the brain stem to limbic and neocortical structures, but it may also represent an association with emerging beta amyloid or tau pathology in these regions.

More recently, the possible roles of iron in determining or affecting clinical phenotypes of PD have been actively explored. Increased QSM values in the medial frontal cortex, anterior cingulate cortex, hippocampus, precuneus, and angular cortex are found in PD patients with anxiety, although such elevation may be related to longer duration of disease in these patients. Regardless, the QSM values of select brain regions were found to be positively correlated with the Hamilton anxiety rating scale (HAM-A) scores [125]. Nigral iron deposition was found to be higher in PD patients with freezing of gait (FOG) than in those without, correlating with the new freezing of gait questionnaire (nFOGQ) scores [112]. Furthermore, elevated iron deposition in the GP has also been associated with akinetic/rigid-dominant PD patients compared to tremor-dominant patients [113]. An extensive number of studies demonstrate the involvement of iron dysregulation in PD pathology. The findings altogether suggest a dynamic nature of iron dysregulation over the disease trajectory. It is important to carefully examine the possible confounding factors contributing to in vivo observations for a better delineation of iron-related mechanisms in PD pathogenesis—including, and not only limited to, patient drug treatment [126]—or acquisition parameters [122].

### 4.2. Cellular Iron Dysregulation in PD

Histological mapping of cellular iron burden in vulnerable anatomic regions of PD post-mortem brains is still missing. The existing studies suggest both glial and neuronal accumulation of iron in disease-affected brains, emphasizing the need for a comprehensive evaluation of pathological iron deposition in different cell types. Administration of α-synuclein pre-formed fibrils to the surface of the olfactory epithelium and olfactory submucosa of *Macaca fascicularis* resulted in robust iron deposition in the SN and the GP, localized to microglia. Such microglial iron burden increased in a time-dependent manner since injection. Moreover, the expression of Tf, TfR, and FPN increased in the dopaminergic neurons, suggesting an increased cycle of cellular iron rather than accumulation [127]. Another study also demonstrated a regional increase in iron in the GP but a decrease in the transferrin/iron ratio in the GP, SN, and caudate nucleus of post-mortem PD brains. Control brains demonstrated relatively more correlated levels, suggesting that pathological iron accumulation assumes the form of NTBI, whose uptake is more favored by the glia than the neurons [128]. Contrastingly, post-mortem studies using X-ray microanalysis coupled with cathodoluminescence spectroscopy and microparticle-induced X-ray emission (uPIXE) demonstrated increased intraneuronal iron in single defined SN neurons in PD [129] and a predominant contribution of dopaminergic neuronal iron burden to R2* MRI signals in the SN [130].

All four hypotheses mentioned above were discussed in terms of PD-related iron pathogenesis; these are summarized here.

### 4.3. Hypothesis 1: Iron Accumulation Is a Consequence of Pathological Alterations in α-Synuclein

In PD, nigral iron accumulation presents as a prominent iron-related disease marker. However, MRI studies examining the changes in SN iron levels throughout disease progression suggest such increase may be more strongly correlated with progression at mid-to-later stages of the disease [122,123,131]. Accordingly, MRI observations of PD nigral iron still report inconsistent results [105,122,126], with even lower levels reported in early stage drug-naïve patients [126], whereas post-mortem examinations consistently report increased iron levels in the SN [102,104,132,133]. Such findings possibly support the hypothesis that iron accumulation follows the local accumulation of α-synuclein pathology. As discussed previously, MRI findings should be interpreted with caution, as even at indifferent levels of observed iron, there may be an unobservable—yet pathogenically meaningful—increase in the levels or a redistribution of iron among the cell populations, which may result in different implications for brain physiology. However, a number of functional studies further support the pathological inducement of iron dysregulation via PD-associated alterations of α-synuclein. As discussed previously, in vivo administration of α-synuclein pre-formed fibrils to the surface of olfactory epithelium and olfactory submucosa of *Macaca fascicularis* resulted in a robust deposition of iron in the SN and GP [127]. The olfactory bulb or the hippocampus and the entorhinal cortex were not affected by abnormal iron deposition. Such observation suggests that PD-associated subcortical iron deposition may represent a regional autonomic factor contributing to the anatomical vulnerability to iron deposition upon accumulation of α-synuclein pathology in the brain. Whether such subcortical iron deposition is a result of—or an inducer of—local α-synuclein pathology is yet to be examined.

Yet, in vitro studies further support the relationship between iron and α-synuclein pathology at the cellular level. Deas et al. demonstrated that the addition of α-synuclein oligomeric species to iPSC-derived SNCA triplication neuronal model resulted in iron-dependent oxidative stress [134]. Disease-related phosphorylation of α-synuclein at Y125 and S129 has been shown to increase its affinity for ferrous iron species [135], which may result in increased sequestration of cellular iron. Moreover, an increase in synuclein protein levels was shown to induce iron dysregulation in dopaminergic cells, potentially via endoplasmic reticulum (ER)-stress-induced changes in iron homeostatic protein expression [136] and in primary midbrain neuronal cultures via increased cellular iron uptake [137].

### 4.4. Hypothesis 2: Iron Accumulation Promotes α-Synuclein Pathology

Biondetti et al. [138] predicted a prodromal phase of 9.6 years for iron deposition in the SN pars compacta (SNc) of PD patient brains, suggesting that iron dysregulation may be involved very early in the disease process. Iron has been shown to regulate α-synuclein expression at the transcriptional and translational levels via IRP–IRE interaction at the 5′-UTR of α-synuclein transcripts. At high levels of cellular iron concentration, active translation of the α-synuclein mRNA is induced [139,140,141,142,143]. At pathological levels of iron, as those observed in MSA and PD, an overexpression of α-synuclein could be expected, which has been implicated with the development of α-synuclein pathology supported by observations that multiplications of the α-synuclein encoding gene (*SNCA*) are associated with genetic forms of PD and dementia with Lewy bodies [144,145,146]. Accordingly, iron chelators as well as IRE inhibitors were shown to decrease the endogenous levels in vitro, and IRP knock-out was shown to increase α-synuclein levels [140,141,147]. Moreover, numerous in vitro studies using recombinant α-synuclein demonstrated the possible role of iron in α-synuclein aggregation via binding of the metal to the protein. Interestingly, studies suggest the involvement of ferric iron species, rather than ferrous species, in the fibrillization process [148,149,150,151]. Ferric iron species has been shown to significantly alter the structure of α-synuclein upon binding from a naturally unfolded to a partially folded form with solvent-exposed hydrophobic patches in a very rapid manner [150]. Ferric iron accelerates and enhances the aggregation process in a dose-dependent manner, as demonstrated by the shorter lag phase and greater maximum signal in ThT assays [151,152]. Ferric-iron-induced intracellular α-synuclein aggregation has been confirmed in HEK293 cells, and further, it has been demonstrated to enhance the transcellular propagation of α-synuclein pathology as well [152]. Importantly, iron-induced aggregated α-synuclein proteins were shown to be species with elevated toxicity, which bind to the A11 antibody, inducing decreased cell viability and formation of pores in bilipid layers [149,151,152]. Furthermore, ferric-iron-induced oligomers demonstrated SDS resistance—like those found in human patients—compared to those formed in the absence of iron. Altogether, the evidence supports the hypothesis that iron-induced α-synuclein aggregates may lead to accumulation in disease-affected human brains.

In addition to direct manipulation of the α-synuclein protein by ferric iron, ferrous iron has been implicated in dose-dependent increases in pathological neuronal α-synuclein alterations, both in vitro and in a *Drosophila* model [153,154], possibly via an underlying interruption of cellular processes, such as autophagy [139,155]. In a dopaminergic neuronal culture from mouse brain SN, ferrous iron treatment increased α-synuclein protein levels in a dose-dependent manner. Moreover, it resulted in an increase in Triton-insoluble α-synuclein levels and their secretion into the culture media by inducing an impairment in autophagosome–lysosome fusion from cellular transcription factor EB (TFEB) redistribution [139]. Ferrous-iron-induced deposition of neuronal α-synuclein pathology via autophagy dysfunction has also been demonstrated in primary neuronal cell culture from SD rats and in SH-SY5Y cells [155]. The authors further describe that sequestration of ROS did not affect the increased α-synuclein levels, suggesting iron, rather than iron-resulting oxidative stress, contributes to α-synuclein pathology. A53T-α-synuclein-mutation-expressing neuroblastoma cells also showed a dose-dependent, ferrous-iron-induced formation of high-molecular-weight α-synuclein aggregates [153]. On the other hand, there is also in vitro evidence suggesting that neuronal oxidative stress contributes to pathological alterations in α-synuclein [140,156,157].

### 4.5. Hypothesis 3: Iron Accumulation Protects from or Hinders the Accumulation of Misfolded Protein Pathology

Interestingly, a study by Dauer Née Joppe et al. [158] demonstrated that injection of α-synuclein pre-formed fibrils into the striatum of neonatal iron-enriched mouse brains resulted in lower levels of P129 α-synuclein and lower α-synuclein pathology in connectome-specific regions. Such observations were not seen in a primary neuronal trans-synaptic protein propagation model, suggesting that iron may alter the connectivity and influence spreading of α-synuclein pathology through an important involvement of glial functions [158].

### 4.6. Hypothesis 4: Iron Dyshomeostasis and Protein Accumulation Are Parallel and Converging Pathways

The evidence demonstrating the reciprocal effect of iron and α-synuclein pathology via multiple molecular and cellular pathways suggests that, regardless of the initial phenomena at disease etiology, once initiated, the accumulation of iron and α-synuclein forms a vicious feedback cycle. This accelerating cycle is expected to develop early in the disease progression, supported by the association of iron dysregulation with early-PD-associated α-synuclein post-modifications [135,136,137]. However, most likely due to the comprehensive involvement of iron and α-synuclein pathology in many cellular processes, their accumulation is not linearly correlated throughout the disease process [97]. Regardless, many studies investigating the relationship between iron and α-synuclein reveal that the accumulation of both is a dual requirement for disease toxicity. First, and as described previously, α-synuclein aggregates formed in the presence of iron exhibit a nature of greater toxicity than those produced in an iron-naïve environment. AAV-vector-treated rat primary neurons (EI4) presented with alterations in intracellular ionic concentrations upon the presence of both α-synuclein overexpression and an iron-enriched environment but not as a result of either alone [137]. Increased vulnerability to iron-induced toxicity has also been demonstrated in human neuroblastoma cells overexpressing α-synuclein compared to those that did not [153]. Moreover, α-synuclein knock-out was shown to prevent iron-, RSL3-, and AA-induced ferroptosis in human midbrain dopaminergic neurons; meanwhile, elevated α-synuclein expression led to an increased vulnerability to lipid peroxidation and ferroptosis [159]. Therefore, iron is suggested to play a critical role in disease toxicity via α-synuclein-mediated mechanisms and inducement of oxidative stress.

### 4.7. Conclusion: Iron Dysregulation in PD Pathogenesis

Among the four hypotheses we define here regarding the relationship between pathological iron and α-synuclein deposition, Hypothesis 2 is most prominently supported by numerous in vitro and in vivo studies. However, studies investigating the functional relationship report multiple dimensions of their relationship (Table 3). Patient MRI observations further support studies addressing the possible synergistic role of iron and α-synuclein in disease toxicity (Hypothesis 4), with reports of correlations of iron levels in α-synuclein-pathology-affected anatomical regions with cognitive or motor dysfunction levels. Altogether, the current evidence suggests a comprehensive role of iron in early disease pathogenesis, possibly in a vicious co-accumulating cycle with α-synuclein, together contributing to elevated toxicity.

## 5. Multiple System Atrophy

MSA is an atypical Parkinsonism, and neuropathologically, a primary oligodendroglial α-synucleinopathy. Synuclein pathology in MSA-affected brains accumulates in oligodendrocytes as glial cytoplasmic inclusions (GCI) and to a lesser extent in neurons as neuronal cytoplasmic and nuclear inclusions (NCI and NNI) [160]. MSA is distinguished into two major disease subtypes, largely based on the predominant clinical symptoms underlined by different vulnerable sites of synuclein pathology and neurodegeneration: (1) the Parkinsonian variant (MSA-P), with predominant striatonigral degeneration (SND), and (2) the cerebellar variant (MSA-C), with olivopontocerebellar atrophy (OPCA) [161,162,163]. More rarely, a third type is identified, associated with frontotemporal dementia and α-synuclein pathology in the frontal and temporal lobes, called atypical MSA or frontotemporal lobar degeneration synuclein variant [164,165]. As mentioned above, MSA-P and MSA-C show different anatomical vulnerability to α-synuclein deposition, mostly distinguished by the initial sites of pathology. In MSA-P, α-synuclein pathology is initially observed to affect the striatum, lentiform nucleus, and the SN, whereas the cerebellum and the brainstem connectivity are observed to be affected earliest in MSA-C [166,167]. Throughout disease progression, overlapping regions become similarly affected by α-synuclein pathology, including the motor cortex, sensory cortex, anterior cingulate gyrus, pyramidal and extrapyramidal white matter, spinal cord, hippocampus, amygdala, and the visual cortex [166,167].

### 5.1. Iron Dysregulation in MSA

Generally, putaminal accumulation of iron has been continuously emphasized in MSA. More recently, such characteristic has been shown to be more distinct in the Parkinsonian subtype of MSA compared to MSA-C. Disease-subtype-specific examination of iron dysregulation reveals distinct patterns of iron deposition in pathology-related anatomical systems (Figure 2). In MSA-P, iron accumulation is consistently most extenuated in the putamen as a disease-distinguishing pathological hallmark [168,169,170]. Moreover, subcortical regions, including the GP and the SN, are affected by greater deposition of iron compared to MSA-C brains [168,170]. Relatively, the putaminal iron levels are low in MSA-C-affected post-mortem and in vivo patient brains, even among the subcortical regions (evaluated in 4 and 28 cases, respectively) [170,171], and they rather demonstrate increased iron deposition in the cerebellum, internal capsule, thalamus, and the dentate nucleus compared to MSA-P groups of similar patient sizes [103,168,170]. Although such iron deposition in the brainstem regions of MSA-C is much less pronounced compared to the putaminal burden in MSA-P, bulk RNA sequencing of 11 post-mortem MSA-C brains revealed significant dysregulation of ferroptosis-related genes, supporting the pathological involvement of iron deposits in these regions [172]. Although correlational evaluations between regional iron depositions with clinical scores are inconsistent [169,173,174], correlation with atrophy has been supported [91,169,175]. In a total of 30 MSA-P cases, the annual change rate in volume and R2* was negatively correlated in the putamen, whereas a negative correlation was observed in the thalamus of 9 MSA-C patients [91,169,175]. The close association of iron accumulation with distinct α-synuclein-pathology-related anatomical regions of the two disease subtypes supports the critical involvement of pathological iron in disease progression and further suggests the two disease subtypes as distinct pathological identities in relation to disease pathogenesis.

### 5.2. Cellular Iron Dysregulation in MSA

In a transgenic mouse model of MSA, increased iron load was observed in the cerebellum at 8 months and in the SN and putamen at 20 months, without any changes in the prefrontal cortex at any time point [176]. In the affected regions, strong Perls staining was observed in the myelinated structures and a subset of myelin-associated cells with eccentric nuclei, which were presumably observed to be oligodendrocytes. Oligodendrocyte-focused iron burden supports the iron–α-synuclein pathology relationship in MSA pathogenesis at the cellular level. However, it is important to note that such cellular iron deposition could also be related to the oligodendrocyte-selective overexpression of α-synuclein in this particular disease model [176]. Following the hypothesis that MSA clinical subtypes have a distinct pathogenesis, including iron-related pathomechanisms, we recently mapped the distinct cellular deposition of pathological iron in the GP, putamen, and the SN in 4 MSA-P- and 4 MSA-C-affected post-mortem human brains [170]. Supporting the hypothesis, the patterns of cellular iron dysregulation in different cell populations were found to be different in the two disease subtypes. In MSA-P, the microglia predominantly accumulate the excess levels of iron consistently throughout the examined regions, whereas in MSA-C, iron is more homogeneously distributed across the glial cell types, as highlighted particularly by the astrocytes accumulating greater or similar levels of iron with microglia. In both disease subgroups, the neurons were least affected by iron deposition and showed a marked absence of any association of abnormal iron deposition with α-synuclein-affected neurons. By comparison, GCIs showed iron deposition in the examined nuclei of both disease subtypes, supporting the possible role of cellular iron accumulation in α-synuclein cytopathology or vice versa [170].

### 5.3. MSA-Specific Evidence of Iron Involvement in Disease Pathogenesis

There are only few studies, which can be discussed within the frame of the aforementioned hypotheses. The pathological relationships between α-synuclein and iron—as explored by numerous in vitro and in vivo studies—were already discussed thoroughly in the previous PD section. Here, we discuss MSA-specific patient-based evidence. Regarding Hypothesis 1, a study by Lee et al. [177] predicting the timing of putaminal iron deposition in MSA disease progression by calculating the conditional probabilities of multiple MRI modalities in 39 probable MSA patients suggested iron deposition as a secondary phenomenon to neurodegeneration, although the relationships with protein pathology using PET were not explored. Regarding Hypothesis 2, it can be mentioned that, on the other hand, the anatomical overlap between abnormal iron accumulation and the initial site of protein pathology, particularly in MSA-P, together with the molecular evidence of iron-induced α-synuclein pathology, as discussed previously, support the possible role of iron in early disease stages. Contrasting with the study by Lee et al. [177], many scholars support the early involvement of iron dysregulation in MSA disease pathogenesis, years prior to clinical onset [178,179,180]. Moreover, there are reports of MSA cases related to genetic alterations in the iron homeostatic ceruloplasmin gene, further supporting the association between early iron dyshomeostasis and MSA [181]. As a contribution of iron to α-synuclein-associated disease toxicity (supportive of Hypothesis 4), the iron levels have been shown to negatively correlate with disease duration in 9 MSA cases [170] and to be implicated with the extent of atrophy and accelerated neurodegeneration in the 39 probable MSA patients [177].

### 5.4. Conclusion: Iron Dysregulation in MSA Pathogenesis

As discussed in the previous PD section, functional studies investigating the relationship between iron and α-synuclein have supported a comprehensive role of iron dysregulation in disease pathology (Table 3). MSA-patient-derived observations are not yet collectively and conclusively supportive of any distinct hypothesis, although the correlation between brain iron burden and atrophy, as well as disease duration, augments the role of iron accumulation in disease toxicity. Based on new insights regarding MSA-subtype-specific iron dysregulation, more subtype-specific patient observations are needed for a comprehensive delineation of the involvement of iron dysregulation in MSA pathogenesis.

## 6. Synthesis and Future Directions

Here, we summarized studies on iron dysregulation in AD, PSP, PD, and MSA at the regional, cellular, and molecular levels, carefully reviewing the literature supporting four possible roles of iron in protein-related disease pathology (Table 4). Although iron accumulation is a common feature across these neurodegenerative diseases, it is evident that the contribution of iron to disease pathology is distinct at all levels of examination. Clinicians treating patients with these neurodegenerative diseases can question the relevance of iron in disease pathogenesis by seeing the lack of effect of currently available iron chelation therapies, such as Deferiprone [182,183]. Such iron chelation therapies target the sequestration of global excess iron. However, and as we demonstrate here, a complete delineation of iron functions in these diseases is challenging due to the different models (post-mortem human sample, animal and cell models, patients) and methods (MRI parameters and output values) used in samples of different disease definitions (clinical biomarkers, disease stages), which makes a cross-study comparison difficult. Heterogenous cytopathologies, distinct regulation mechanisms of iron, pathological proteins, and their interaction in different cell types of the human brain require more investigations of their association at the cellular and cell-type-specific level for further delineation of iron functions in disease pathogenesis. Further use of high-resolution quantitative measurement techniques, such as X-ray spectromicroscopy and inductively coupled plasma mass spectrometry (ICP-MS), would likewise enhance our understanding of neurodegenerative pathological iron deposition. Moreover, the dynamic nature of these interactions highlights the need for a more systematic design of MRI studies in combination with biomarker testing of the patients involved, such as PET or real-time quaking-induced conversion (RT-QuIC) [184], for delineation of iron dysregulation over the course of disease. Based on these findings, we propose that the heterogeneity and complexity of iron involvement in these diseases demonstrate the need for more intricate disease-specific iron targets or strategies for more defined patient sub-cohorts for better clinical outcomes. Second-generation iron chelation therapies, which involve a combinational strategy targeting iron with other players in inflammatory pathways, may provide a more promising direction [182].

## Figures and Tables

**Table 1:** Summary of AD-related in vitro and in vivo findings directly addressing the defined hypotheses between iron, Aβ, and tau: (1) Iron accumulation is a consequence of pathological alterations related to protein pathology; (2) Iron accumulation promotes protein pathology; (3) Iron accumulation protects from or hinders the accumulation of misfolded protein pathology; and (4) Iron dyshomeostasis and protein accumulation are parallel and converging pathways. Abbreviations: Alzheimer’s disease (AD), amyloid-beta (Aβ), wild-type (WT), knock-out (KO).

Hypothesis	Study	Sample Model	Relevant Findings
Hypothesis 1 (Aβ)	Dekens et al. [48]	Primary astrocytic culture	Addition of human recombinant Aβ in culture medium resulted in increased ferritin expression.
	Baringer et al. [39]	iPSC-derived endothelial cells and astrocytes	Aβ exposure led to increased iron uptake by astrocytes and iron transport across the blood–brain barrier model of endothelial cells.
	McIntosh et al. [50]	Primary microglial culture and APPswe/PS1dE9 mice	IFNγ and Aβ exposure led to increased glial iron retention.
	Li et al. [51]	WT C57/B16 mice	Intrahippocampal injection of Aβ oligomers induced increased APP expression and iron accumulation.
	Tsatsanis et al. [46]	Primary neuronal culture	Inhibition of α-secretase activity led to increased cellular iron retention.
Hypothesis 1 (tau)	Lei et al. [42]	Primary neuronal culture and Tau KO C57BL/6/SV129 mice	Tau KO led to cellular iron retention and iron accumulation in the cortex, hippocampus, and the substantia nigra.
Hypothesis 2 (Aβ)	Banerjee et al. [58]	SHSY5Y cells and monomeric Aβ42	Ferric ammonium citrate (FAC) treatment induced increased β-secretase activity and Aβ42 secretion, and oligomerization of monomeric Aβ42.
	Chen et al. [59]	Primary neuronal culture	FAC treatment induced abnormal cellular localization of soluble APP α.
	Becerill-Ortega et al. [60]	Primary neuronal and astrocytic culture and APP/PS1 mice	Iron exposure led to neuronal-specific increase in KPI-APP (pro-amyloidogenic form) expression and secretion of Aβ42.
	Chen et al. [61]	APP/PS1 mice	Dietary iron treatment led to increased Aβ in the hippocampus.
	Tahmasebinia and Emadi [62]	Aβ40 and Aβ42 peptides	Ferric chloride treatment promoted the aggregation of Aβ40 and Aβ42 peptides.
Hypothesis 2 (tau)	Wan et al. [66]	Primary neuronal culture and C57BL/6 mice	Iron treatment led to increased phosphorylation at Ser202/Thr205, Thr181, and Ser396 sites of tau.
	Yamamoto et al. [68]	PHF-tau from post-mortem AD tissue	Ferric iron bound to hyperphosphorylated tau and induced aggregation in a dose-dependent manner.
	Ahmadi et al. [69]	Tau-410	Ferrous and ferric iron bound to tau and induced structural changes.
	Guo et al. [67]	APP/PS1 mice	Iron-rich diet resulted in increased phosphorylation of tau at Thr205, Thr231, and Ser396 sites.
Hypothesis 3	-	-	-
Hypothesis 4	-	-	-

**Table 2:** Summary of PSP-related in vitro and in vivo findings directly addressing the defined hypotheses between iron and tau: (1) Iron accumulation is a consequence of pathological alterations related to tau pathology; (2) Iron accumulation promotes tau pathology; (3) Iron accumulation protects from or hinders the accumulation of misfolded tau pathology; and (4) Iron dyshomeostasis and tau accumulation are parallel and converging pathways. Abbreviations: Progressive supranuclear palsy (PSP).

Hypothesis	Study	Sample Model	Relevant Findings
Hypothesis 1	Please refer to Table 1, rows under “Hypothesis 1 (tau)”.		
Hypothesis 2	Mukherjee and Panda [96]	4R2N recombinant tau	Ferric iron induced tau oligomerization and fibrillization in a dose-dependent manner.
	For more information, please refer to Table 1, rows under “Hypothesis 2 (tau)”.		
Hypothesis 3	-	-	-
Hypothesis 4	-	-	-

**Table 3:** Summary of in vitro and in vivo findings directly addressing the defined hypotheses between iron and α-synuclein: (1) Iron accumulation is a consequence of pathological alterations related to α-synuclein pathology; (2) Iron accumulation promotes α-synuclein pathology; (3) Iron accumulation protects from or hinders the accumulation of misfolded α-synuclein pathology; and (4) Iron dyshomeostasis and α-synuclein accumulation are parallel and converging pathways. Abbreviations: α-synuclein (α-syn), globus pallidus (GP), substantia nigra (SN), knock-out (KO).

Hypothesis	Study	Sample Model	Relevant Findings
Hypothesis 1	Guo et al. [127]	*Macaca fascicularis*	Administration of pre-formed α-syn fibrils resulted in robust iron deposition in the SN and GP, localized to microglia, and altered iron homeostatic protein expression in dopaminergic neurons.
	Deas et al. [134]	iPSC-derived neuron with SNCA triplication mutation	Exposure to exogenous α-syn oligomeric species resulted in iron-induced oxidative stress.
	Mi et al. [136]	MES23.5 dopaminergic cells	Addition of recombinant α-syn to culture media induced dysregulation of iron homeostatic genes.
	Ortega et al. [137]	Primary neuronal culture	Overexpression of human α-syn in an iron-rich environment resulted in increased intracellular iron retention.
Hypothesis 2	Zhao et al. [148]	Human recombinant α-syn	Incubation with ferric iron promoted α-syn fibrillization at low concentrations.
	Abeyawardhane et al. [149]	Human recombinant α-syn	Under aerobic conditions, ferrous iron incubation induced a soluble α-syn oligomer structure, and ferric iron induced a fibril structure with β-sheets, both of elevated toxic species.
	Uversky et al. [150]	Human recombinant α-syn	Incubation with ferric iron induced accelerated and increased aggregation of α-syn.
	Kostka et al. [151]	Human recombinant α-syn	Incubation with ferric iron enhanced α-syn aggregation, producing larger and toxic oligomers.
	Li et al. [152]	Human recombinant α-syn and HEK293 cells	Incubation with ferric iron enhanced the seeded aggregation of α-syn in a dose-dependent manner, demonstrating enhanced intracellular aggregation and transcellular propagation of α-syn; iron-seeded fibrils were more cytotoxic.
	Ostrerova-Golts et al. [153]	Human BE-M17 neuroblastoma cells transfected with WT, A53T, A30P α-syn	Treatment with ferrous iron induced a dose-dependent formation of high-molecular-weight α-syn aggregates in A53T α-syn-expressing cells.
	Agostini et al. [154]	α-syn-overexpressing dopaminergic neuron Drosophila model	Administration of ferric ammonium citrate accelerated α-syn pathology formation in dopaminergic neurons and reduced fly life span.
	Xiao et al. [139]	Dopaminergic cell line SN4741	Ferrous iron promoted α-syn aggregation and secretion via inhibition of autophagosome–lysosome fusion.
	Wang et al. [155]	Sprague Dawley rats and SH-SY5Y cells	Iron upregulated α-syn phosphorylation at Ser129 and high-molecular-weight-α-syn levels in the SN and dopaminergic cells.
	Li et al. [140]	SK-N-SH cells	Ferrous and ferric iron induced cell loss and α-syn aggregation, both directly and via oxidative stress.
Hypothesis 3	Dauer Née Joppe et al. [156]	C57BI/6J mice and primary neuronal culture	Neonatal iron-enriched mice showed significantly less α-syn pathology in connectome-specific regions.
Hypothesis 4	Ortega et al. [137]	Primary neuronal culture	Overexpression of α-syn in an iron-rich environment resulted in increased intracellular iron retention, which could not be induced via either α-syn overexpression or iron enrichment alone.
	Osterova-Golts et al. [153]	Human BE-M17 neuroblastoma cells transfected with WT, A53T, A30P α-syn	Cells overexpressing all types of α-syn were more vulnerable to iron-induced toxicity.
	Mahoney-Sanchez et al. [159]	LUHMES cells (human dopaminergic neuronal model)	α-syn KO prevented ferroptosis.

**Table 4:** Summary of current evidence on AD, PSP, PD, and MSA supporting the following hypotheses (for reference, please see the disease-specific sections in the text): (1) Iron accumulation is a consequence of pathological alterations in protein; (2) Iron accumulation promotes protein pathology; (3) Iron accumulation protects from or hinders the accumulation of misfolded protein pathology; and (4) Iron dyshomeostasis and protein accumulation are parallel and converging pathways. Representative colors: Gray = Absence of supporting evidence/not examined; Light blue = Weakly supported/limited supporting evidence (especially due to scarcity of data on human studies focusing on PSP and MSA); Blue = Moderately supported; Dark blue = Strongly supported. Abbreviations: Alzheimer’s disease (AD), amyloid-β (Aβ), progressive supranuclear palsy (PSP), Parkinson’s disease (PD), multiple system atrophy (MSA), magnetic resonance imaging (MRI), post-mortem studies (PM), animal model studies (AM), in vitro studies (IV).

	Hypothesis 1				Hypothesis 2				Hypothesis 3				Hypothesis 4			
	MRI	PM	AM	IV	MRI	PM	AM	IV	MRI	PM	AM	IV	MRI	PM	AM	IV
**AD (** **A** **β** **)**																
**AD (tau)**																
**PSP**																
**PD**																
**MSA**																
